# Modeling the Transitions between Collective and Solitary Migration Phenotypes in Cancer Metastasis

**DOI:** 10.1038/srep17379

**Published:** 2015-12-02

**Authors:** Bin Huang, Mohit Kumar Jolly, Mingyang Lu, Ilan Tsarfaty, Eshel Ben-Jacob, Jose’ N Onuchic

**Affiliations:** 1 Center for Theoretical Biological Physics, Houston, TX 77005-1827, USA; 2 Department of Chemistry, Houston, TX 77005-1827, USA; 3 Department of Bioengineering, Houston, TX 77005-1827, USA; 4 Department of Physics and Astronomy, Houston, TX 77005-1827, USA; 5 Department of Clinical Microbiology and Immunology, Sackler School of Medicine Tel-Aviv University, Tel-Aviv 69978, Israel; 6 Department of Biosciences, Rice University, Houston, TX 77005-1827, USA; 7 School of Physics and Astronomy and The Sagol School of Neuroscience Tel-Aviv University, Tel-Aviv 69978, Israel

## Abstract

Cellular plasticity during cancer metastasis is a major clinical challenge. Two key cellular plasticity mechanisms —Epithelial-to-Mesenchymal Transition (EMT) and Mesenchymal-to-Amoeboid Transition (MAT) – have been carefully investigated individually, yet a comprehensive understanding of their interconnections remains elusive. Previously, we have modeled the dynamics of the core regulatory circuits for both EMT (miR-200/ZEB/miR-34/SNAIL) and MAT (Rac1/RhoA). We now extend our previous work to study the coupling between these two core circuits by considering the two microRNAs (miR-200 and miR-34) as external signals to the core MAT circuit. We show that this coupled circuit enables four different stable steady states (phenotypes) that correspond to hybrid epithelial/mesenchymal (E/M), mesenchymal (M), amoeboid (A) and hybrid amoeboid/mesenchymal (A/M) phenotypes. Our model recapitulates the metastasis-suppressing role of the microRNAs even in the presence of EMT-inducing signals like Hepatocyte Growth Factor (HGF). It also enables mapping the microRNA levels to the transitions among various cell migration phenotypes. Finally, it offers a mechanistic understanding for the observed phenotypic transitions among different cell migration phenotypes, specifically the Collective-to-Amoeboid Transition (CAT).

Metastasis causes more than 90% of cancer-related deaths^1^. For carcinomas, the most common type of tumors, metastasis begins when some epithelial cells from the primary tumor lose their apico-basal polarity and cell-cell adhesion and acquire migratory and invasive characteristics, through a process known as **E**pithelial–to-**M**esenchymal **T**ransition (EMT)^2^. Cells can undergo a partial or complete EMT and consequently move collectively or individually while treading through the extra-cellular matrix (ECM) and circulating in the bloodstream^3^,^4^. Upon reaching the secondary site, these circulating tumor cells (CTCs) exit the bloodstream and usually undergo a Mesenchymal-to-Epithelial Transition (MET) to seed metastases^2^.

The collectively migrating cells display both epithelial (E) (cell-cell adhesion) and mesenchymal (M) (migration) properties, thereby reflective of the hybrid epithelial/mesenchymal (E/M) or partial EMT phenotype^4^; while the individually moving cells display at least two distinct phenotypes—amoeboid (A) and mesenchymal (M). Cells in the M phenotype, i.e. the ones that do undergo a complete EMT, secrete Matrix Metalloproteinases (MMPs) to remodel and degrade the ECM, therefore acting as ‘path generators’^5^,^6^. Conversely, cells in the A phenotype do not secrete MMPs, rather squeeze into the gaps in the ECM and migrate as ‘path finders’^5^,^6^. Cancer cells can switch from A to M phenotype or vice-versa by undergoing an **A**meoboid-to-**M**esenchymal **T**ransition (AMT) or a **M**esenchymal-to-**A**moeboid **T**ransition (MAT)^7^ spontaneously or under the influence of external microenvironment^8^,^9^,^10^. Recent studies have identified several individually migratory phenotypes displaying mixed amoeboid and mesenchymal characteristics^7^,^11^,^12^,^13^, indicative of a hybrid amoeboid/mesenchymal (A/M) phenotype^14^. During metastasis, cells can often switch among these different modes of migration. Such rich plasticity allows cancer cells to adapt to the changing microenvironment quickly and facilitates tumor metastasis^2^,^3^,^4^,^5^.

Although the mechanisms of EMT/MET^2^,^15^,^16^ and MAT/AMT^5^,^14^ are well studied individually, a comprehensive understanding of how EMT/MET and MAT/AMT are connected remains elusive. Collectively migrating cells in E/M phenotype can switch to individually migrating cells in M phenotype or *vice-versa* during EMT^4^. Little is known, however, on how E/M cells undergo a **C**ollective-to-**A**moeboid **T**ransition (CAT). CAT has been specifically observed in a cluster of migrating melanoma cells^17^ and in the invasion of fibrosarcoma cells^18^. Therefore, deciphering the operating principles of the inter-conversion between the collective and the individual modes of migration would be crucial to develop anti-metastasis therapies.

Our previous theoretical work has explained how the core EMT/MET regulatory circuit allows transitions between E/M phenotype displaying collective cell migration and the mesenchymal (M) phenotype displaying individual migration^15^. The core regulatory circuit consists of two interconnected mutually inhibitory circuits between a microRNA and a transcription factor (TF) – miR-34/SNAIL and miR-200/ZEB^4^ (Fig. 1a). miR-34/SNAIL acts as an integrator of various external signals for inducing or inhibiting EMT, and feeds to miR-200/ZEB that acts as the three-way decision making switch for EMT/MET, thereby allowing for three distinct phenotypes – E (high miR-200, low ZEB), M (low miR-200, high ZEB) and E/M (medium miR-200, medium ZEB)^15^. Also, our previous work has elucidated how the core AMT/MAT regulatory circuit enables for transitions among the three modes of individual migration – A, M and hybrid A/M^14^. The core regulatory network is the mutually inhibitory circuit between two GTPases – RhoA and Rac1 – that inhibit the GTP loading of one another^5^ (Fig. 1a). High levels of active RhoA (or RhoA-GTP) correspond to amoeboid phenotype (A)^5^, high levels of active Rac1 (or Rac1-GTP) correspond to mesenchymal phenotype (M)^5^, and both high levels of active Rac1 and RhoA correspond to hybrid amoeboid/mesenchymal (A/M) phenotype^14^.

Understanding the operating principles of inter-conversion between collective (E/M) and these different individual modes of migration (A, A/M, M) requires analyzing the coupling of core regulatory circuits of EMT/MET and AMT/MAT. These two regulatory circuits are coupled through the microRNAs, miR-200 and miR-34, that inhibit both RhoA as well as Rac1^19^,^20^,^21^,^22^,^23^,^24^ Fig. 1a. Therefore, the coupled circuit contains links that are both translational (through microRNAs) and post-translational (through GTPases) in nature. These interactions can potentially give rise to novel dynamical features that are not observed in circuits composed of only transcription factors, or those with both transcription factors and microRNAs^15^,^25^. This inherent complexity in the coupled system explains why the transitions between collective and solitary migration phenotypes have received limited theoretical attention.

Here, we follow and extend our previous theoretical framework to study small GTPase-based regulations in RhoA/Rac1 circuit by adding microRNA-mediated inhibition on the production of RhoA and Rac1. We show that this coupled circuit can introduce another stable steady state in addition to the three stable steady states (A, A/M, M) for the standalone Rac1/RhoA circuit^14^. We propose to associate this extra stable state with the hybrid epithelial/mesenchymal (E/M) phenotype, a phenotype that enables collective cell migration. We also found that the cells moving collectively (E/M) can switch to being in either an amoeboid (A) or a mesenchymal (M) phenotype directly, depending on the relative strength of inhibition of RhoA and Rac1 by the microRNAs. Our results further indicate that the high levels of microRNAs, miR-200 and miR-34, can restrict the transitions towards solitary migration phenotypes, even in the presence of EMT-stimulating signals, such as Hepatocyte Growth Factor (HGF). Our model is consistent with several experimental observations and provides the first step towards understanding how different levels of miR-200 and miR-34, and the Rho GTPases in a cell govern phenotypic plasticity during carcinoma metastasis under the influence of external signals in the tumor microenvironment.

## Results

### Mathematical Model of the Coupled Circuit

**Figure 1:**
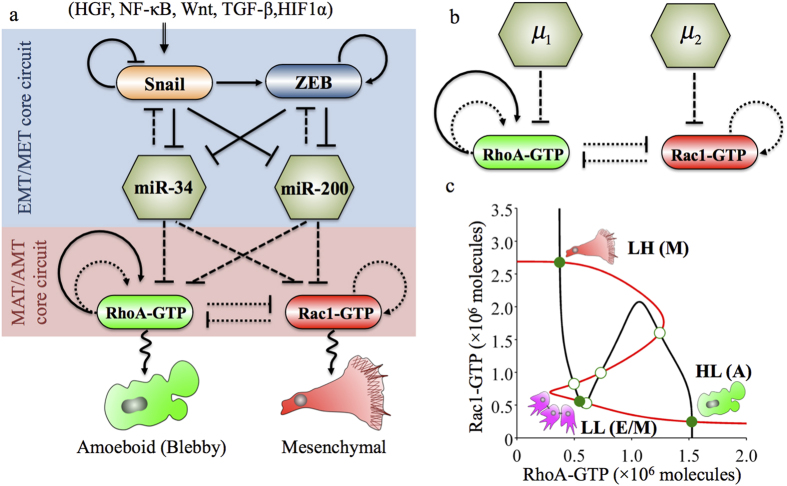
The association between the core regulatory circuits for EMT/MET and MAT/AMT. (**a**) The core EMT/MET regulatory circuit consists of two coupled mutually inhibitory circuits (SNAIL/miR-34 and ZEB/miR-200). It can receive external EMT-inducing signals such as HGF, and regulate the Rac1/RhoA circuit by inhibiting the translation of RhoA and Rac1 via miR-34 and miR-200. (**b**) The effective (reduced) circuit, where *μ*_1_ represents the inhibition on RhoA by either miR-34 or miR-200, and *μ*_2_ represents similar inhibition on Rac1. A solid arrow denotes activation, and a solid bar denotes repression. A solid line represents transcriptional regulation, a dotted line represents indirect regulations on GTP loading or hydrolysis process via GEFs or GAPs, and a dashed line represents translational inhibition by microRNAs (**c**) Dynamical system characteristics of the effective circuit. The plot shows the nullclines and possible steady states corresponding to equation (1). When *μ*_1_ = *μ*_2_ = 100 molecules, the circuit can be tri-stable (LH, LL, HL). Red nullcline is for 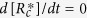 and black nullcline is for 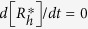. Green solid circles denote the stable steady states, and green hollow circles denote the unstable steady states. Each stable state can be associated with a cell phenotype, as depicted by a cartoon beside them.

The EMT regulatory circuit regulates the Rac1/RhoA circuit via miR-34 and miR-200 Fig. 1a. Here, we use an effective (reduced) model (Fig. 1b) to represent the association between these two circuits – miR-34/SNAIL/miR-200/ZEB (the core circuit for EMT/MET) and RhoA/Rac1 (the core circuit for AMT/MAT) (Fig. 1a). As shown in Fig. 1b, the effect of EMT regulatory circuit is treated as two external signals to the Rac1/RhoA circuit, where (*μ*_1_) represents the inhibition on RhoA by either of the two microRNAs (miR-34 or miR-200) while (*μ*_2_) represents a similar inhibition on Rac1.

We have previously developed the theoretical framework for small GTPase-based regulatory circuit such as Rac1/RhoA circuit^14^, as well as that for microRNA-based regulatory circuit like miR-34/SNAIL and miR-200/ZEB^15^. Here, we use both these frameworks to study the dynamics of the coupled effective circuit. The deterministic dynamics of the circuit can be modeled by two rate equations as given below:


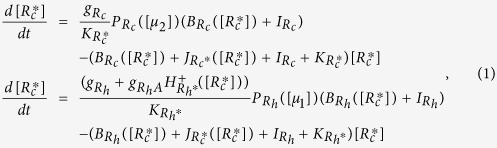


where 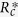 represents the active Rac1 (Rac1-GTP), and 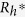 represents the active RhoA (RhoA-GTP). 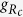
is the production rate for Rac1, 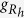
and 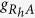 are basal and excitatory production rates for RhoA respectively. 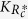 and 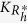 are the corresponding degradation rates for Rac1 and RhoA. 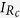 and 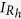 represent two external signals that drive the GTP hydrolysis/loading process of the Rac1/RhoA circuit, such as Grb2 and Gab1 in c-MET signaling, whose effects have been analyzed in our previous work^14^. The Hill function 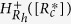 represents the transcriptional self-activation of RhoA. B and J functions are defined to represent the total GTP loading and hydrolysis rates including the intrinsic ones for RhoA and Rac1 and also the activated ones by GEFs or GAPs (See Supplementary Information section 1)^14^. The inhibition effect of microRNAs is described by the function *P*(*[μ*]) whose value ranges from 0 to 1 – 0 denotes a strong silencing effect, and 1 denotes no effect^15^. This effective model (eq. (1)) was used for stability and bifurcation analyses while the detailed model shown in Supplementary Information section 1 was used for the dynamic simulations below. The model derivation and the parameter estimations are described in details in the Supplementary Information.

### The additional stable steady state corresponding to collective migration phenotype

As reported before, the standalone Rac1/RhoA circuit (i.e. without any input from miR-34 and miR-200) can act as a three-way switch enabling these three states: (a) (high active RhoA/low active Rac1), denoted by (HL, or (1, 0)), (b) (low active RhoA/high active Rac1) denoted by (LH, or (0+, 1)) and (c) (both high active RhoA and Rac1) denoted by (HH, or (1, 1)). Based on experimental observations^7^,^11^,^12^,^13^, these states can be associated with the amoeboid (A), mesenchymal (M) and hybrid amoeboid/mesenchymal (A/M) phenotypes respectively^14^.

Now, considering the connections between Rac1/RhoA circuit and EMT regulatory circuit, i.e. in presence of low levels of microRNAs (miR-34 and miR-200), the system can display another stable state that has relatively low levels of both active RhoA and Rac1, as denoted by (LL, or (0, 0)) (Fig. 1c). At certain levels of microRNAs, the system can also behave as a four-way switch such that all these steady states—LL, HL, HH, and LH – co-exist (Supplementary Fig. S1). We hereby propose to associate this new (LL) state with the hybrid epithelial/mesenchymal (E/M) phenotype. This association is corroborated by experimental observations of the presence of balanced, moderate levels of active Rac1 and RhoA in cells of the hybrid E/M phenotype not only during cancer metastasis (type III EMT), but also during wound healing (type II EMT), and embryonic development (type I EMT)^26^,^27^,^28^.

### The RhoA/Rac1 Circuit Response to microRNA Signals

**Figure 2:**
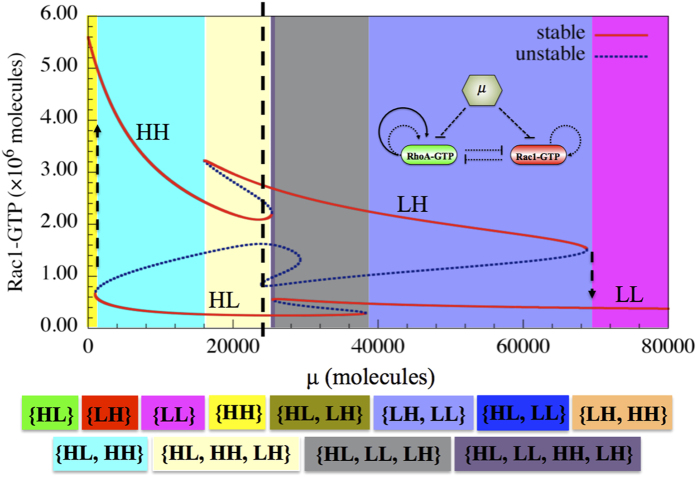
Bifurcation of Rac1-GTP protein levels in response to microRNAs (μ) signal regulating the translation of Rac1 and RhoA. Red solid lines mark the stable states, and blue dashed lines represent unstable states. The bifurcation illustrates the possible co-existence (for some range of the signal) of four states (LL, LH, HH, and HL). The corresponding bifurcation of RhoA-GTP protein levels is shown in Supplementary Fig. S3. The co-existence of different phenotypes is associated with a multi-stable phase, highlighted by different background colors (legends given at the bottom of the figure). This bifurcation also indicates the possible state transitions when signal μ changes. Starting from the HL state (A phenotype, the red line at bottom left part of the diagram), the system could undergo a transition to the HH state (A/M phenotype, the red line at top left part of the diagram) when μ signal decreases. The transition is indicated by the dashed upward arrow at the boundary of the phase {HL, HH} (cyan region) and {HH} (yellow region). Similarly, starting from LH state, the increasing μ signal can induce the transition from the LH state (M phenotype) to the LL state (E/M phenotype), as indicated by the downward arrow at the boundary of the phase {LL} (pink area) and {LL, LH} (light blue region).

Next, as the first step towards analyzing the response of the Rac1/RhoA circuit to different levels of the external microRNA signals (*μ*_1_ and *μ*_2_), we consider *μ*_1_ and *μ*_2_ to be equal at all times; and that allows us to consider them together as a single external signal denoted by μ (Fig. 2). Different levels of the signal μ enable different sets of co-existing phenotypes and hence different multi-stable phases, each marked by a distinct color in the figure. At a high level of μ, cells have relatively lower activities of both RhoA and Rac1, and can thereby adopt only the LL state, i.e. a hybrid E/M phenotype (marked by the pink region). As the level of microRNAs decreases, cells may undergo the transition to adopt a LH state (M phenotype) or a HL state (A phenotype) either deterministically or stochastically. With further decrease in the levels of μ, cells can attain relatively high levels of both active RhoA and active Rac1, thereby adopting the HH state (A/M phenotype) state. Our results are consistent with experiments showing that miR-34 and miR-200 can act as gatekeepers of metastasis and that their decrease leads to collective as well as individual migration^29^,^30^.

### Two-parameter Bifurcation Diagram

**Figure 3:**
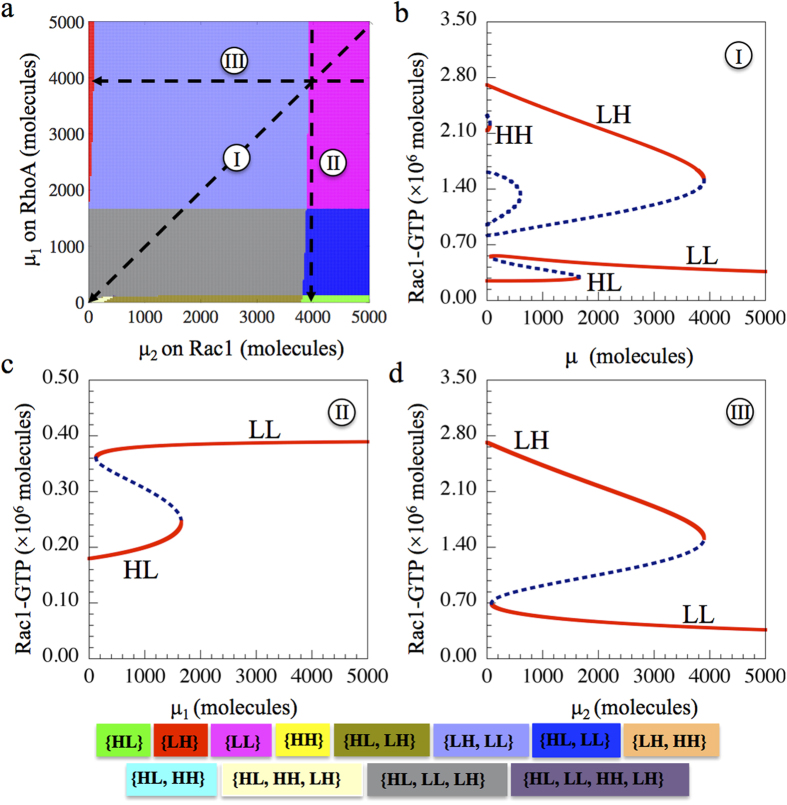
The circuit response to the input signals *μ*_1_ and *μ*_2_. (**a**) Two-parameter bifurcation phase diagram; where the two bifurcation parameters are the two input signals *μ*_1_ (y-axis) and *μ*_2_ (x-axis). Each phase, marked by a distinct color (shown in bottom), corresponds to a different combination of coexisting phenotypes (Phase plane diagrams for each phase are showed in Supplementary Fig. S4). (**b**) One-parameter bifurcation diagram when both *μ*_1_ and *μ*_2_ change simultaneously at the same rate, allowing them to merge into one parameter (here it is referred to as μ), as shown by the trajectory I in the two-parameter bifurcation phase diagram (Fig. 3a). (**c**) One-parameter bifurcation diagram for the circuit driven by varying levels of *μ*_1_ for a fixed *μ*_2_ = 4,000 molecules, corresponding to the trajectory II in the two-parameter bifurcation phase diagram (Fig. 3a). (**d)** One-parameter bifurcation diagram for the circuit driven by varying levels of *μ*_2_ at a fixed *μ*_1_ = 4,000 molecules, corresponding to the trajectory III in the two-parameter bifurcation phase diagram (Fig. 3a).

Next, we analyze the response of the circuit when the two microRNA signals (*μ*_1_ and *μ*_2_) can change independently (Fig. 3a). We considered the initial condition such that the cells can adopt any of the three individually migrating phenotypes – A, M, and A/M (marked by black dashed line in Fig. 2 where μ = 25,000 molecules). At different levels of these two signals (*μ*_1_ and *μ*_2_), one or several different states or phenotypes can co-exist to form different phases. More specifically, the possible phases are: 1. Phases with only one state – {HL}, {LH} and {HH}. 2. Phases in which two states can coexist – {HL, LL}, {LH, LL} and {HL, LH}. 3. A phase in which three states can coexist – {HL, LH, LL} and {HL, LH, HH}. 4. A phase in which all four states can coexist – {HL, LH, LL, HH}. These various phases indicate the plasticity of cell migration phenotypes as driven by different combinations of microRNAs (*μ*_1_ and *μ*_2_ signals).

Depending on how *μ*_1_ and *μ*_2_ change temporally, the cells follow different trajectories in this phase diagram and thus go through different phenotypic transitions as is illustrated in Fig. 3b–d. When both the microRNAs – one inhibiting RhoA and the one inhibiting Rac1 – are decreased at the same rate, we see the co-existence of all three solitary migration phenotypes—M, A/M and A phenotypes, i.e. plasticity of cell migration phenotypes is quite rich (Fig. 3b). However, if only one of the microRNAs decreases, cells finally adopt either A or M phenotype (Fig. 3c,d).

### Temporal Dynamics

**Figure 4:**
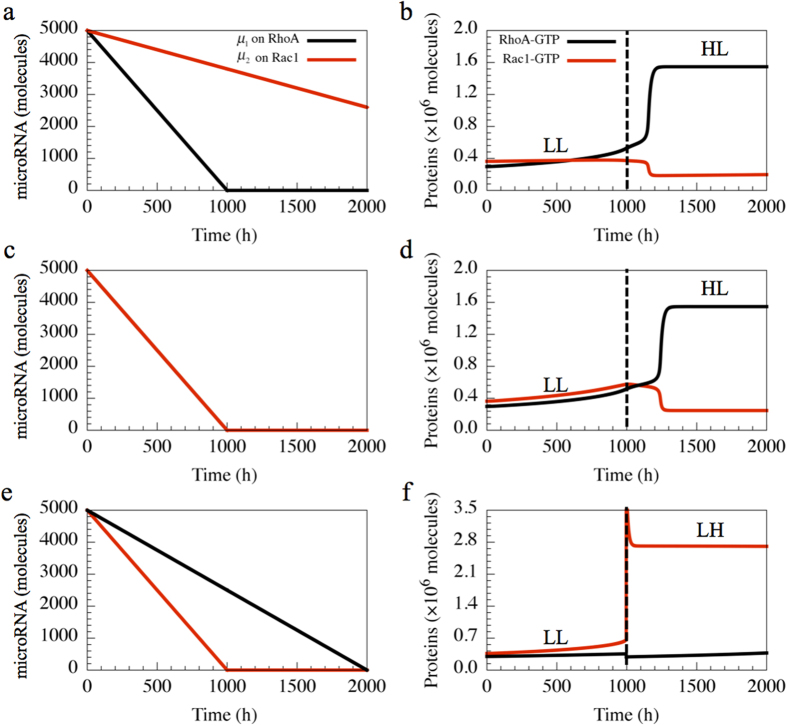
Temporal dynamics of the circuit in response to different rates at which *μ*_1_ and *μ*_2_ decrease. When *μ*_1_ decreases faster than *μ*_2_ (**a**), the cell undergoes a transition from the LL to the HL state (**b**). When *μ*_1_ and *μ*_2_ decrease at the same rate **(c)**, cell still switches from the LL to the HL state **(d)**. When *μ*_1_ decreases slower than *μ*_2_ **(e)**, the cell switches to the LH state from the LL state **(f)**.

We demonstrate the temporal dynamics of a phenotypic transition of a cell from collective migration (E/M phenotype) to individual migration (A, M and A/M phenotypes). In the E/M phenotype (LL state), cells have high levels of microRNAs (*μ*_1_ and *μ*_2_). When these signals decrease, cells gradually gain the ability to migrate and can eventually start moving individually in one of the solitary migration phenotypes—A, M and A/M. Notably, in different biological contexts, the two microRNAs (the one inhibiting RhoA (*μ*_1_) and the other inhibiting Rac1 (*μ*_2_)) may decrease at different rates, thus leading to a difference in the dynamics of phenotypic transition. As shown in Fig. 4, if the microRNA inhibiting RhoA (*μ*_1_) decreases faster than the microRNA inhibiting Rac1 (*μ*_2_) (Fig. 4a,b), the cells transit from the E/M phenotype to A phenotype, i.e. they undergo direct collective to amoeboid transition (CAT) as observed in fibrosarcoma cells^18^. Similar behavior is observed when both microRNAs decrease at same rate (Fig. 4c,d). In contrast, when (*μ*_2_) decreases faster (Fig. 4e), E/M to M phenotype transition may occur (Fig. 4f), i.e. the cells undergo a complete EMT.

### Effective Landscape

In order to understand the relative stability of the co-existing stable states (phenotypes), we construct the effective landscape by considering the biological noise that can be present due to fluctuations in gene expression. Noise can originate from many sources such as birth/death of species and binding/unbinding of proteins, and can induce switches among its stable steady states spontaneously without the action of any external signal. The effective potentials of the landscape are defined as the negative logarithm of the probability (P) of each state (−ln(P))^31^,^32^. Therefore, the more common (or frequently adopted) steady state (or phenotype) would have a lower effective potential as shown by blue color. The larger the blue area surrounds a steady state, the more frequently the state or the phenotype is observed.

**Figure 5:**
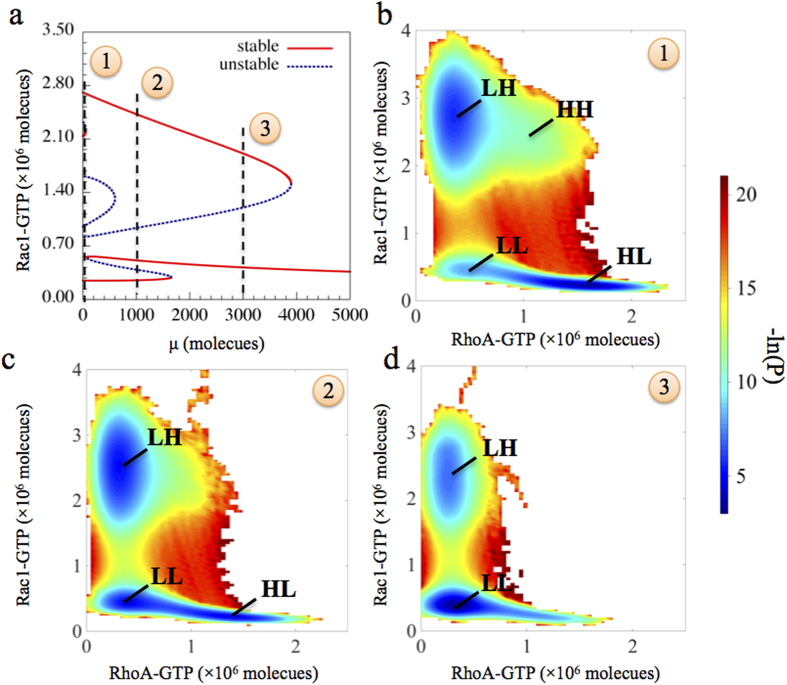
Effective landscapes of the circuit with Gaussian white noise. (**a**) Bifurcation diagram of the stable Rac1-GTP levels in response to different level of μ. Three different levels of μ are highlighted, as they are the conditions to calculate the effective landscape values (-ln(P)) by Langevin simulation. (**b–d**) show effective landscapes for (**b)** μ = 0, **(c)**: μ = 1000, and **(d)** μ = 3000 (all units in molecules). The basins associated with each state is labeled for each case. The larger the blue region surrounding a particular steady state, the more frequent the state is.

Here, we have simulated Langevin dynamics of our model by adding Gaussian white noise (see the method section for details) for three cases (Fig. 5a), each of which corresponds to a different level of μ. Without this inhibition signal (μ), four steady states (HL, LH, HH, LL) can be detected. Notably, without the effect of noise, i.e. in the deterministic analysis, the system had three stable states (HL, LH, HH) (Fig. 1), but noise can induce transitions to another steady stable state (LL). The states representing the A, E/M and M phenotypes are relatively more frequently observed than the state representing the state of the A/M phenotype, as indicated by the higher effective potentials for the HH basin (Fig. 5b). Also, we observed more transitions between the A and E/M or M and E/M, rather than the other possible transitions (Supplementary Fig. S7a). However, with increasing levels of μ, the HH state disappears and the system could still stay in the HL, LH or LL states, but the probability of staying in the LL state increases while that for the LH and the HL states decreases (Fig. 5c and Supplementary Fig. S7b). For even higher levels of μ, the transitions from the collective to the solitary migration is largely inhibited (Fig. 5d and Supplementary Information section 7) therefore indicating that microRNAs can potentially stabilize the E/M phenotype against the biological noise.

### The switch responds to the input signals from c-Met pathway at different microRNA levels

Until now, we have considered the effect of microRNAs on the AMT/MAT circuit in the absence of any EMT-inducing signal. Here, we analyze the response of the AMT/MAT switch or circuit in the presence of an EMT-inducing signal – the HGF/c-Met pathway. c-MET is a tyrosine kinase receptor that is encoded by an oncogene, and is activated when the signaling molecule HGF binds to it^33^. The downstream effectors of the c-MET/HGF pathway include Grb2 and Gab1 that can regulate cell migration phenotypes by activating RhoA or Rac1^34^,^35^,^36^,^37^,^38^ respectively.

To consider the regulatory functions of all these signals combined together, we first calculated the two-parameter bifurcation diagrams (See Supplementary Information section 8) when the Rac1/RhoA regulatory circuit is driven by two of these signals – (a) μ and Grb2 (Supplementary Fig. S8a), and (b) μ and Gab1 (Supplementary Fig. S8b). As expected, high levels of microRNAs suppress the stimulation of solitary migration by Grb2 and Gab1 signals and therefore retain the cells in the LL or hybrid E/M phenotype (Supplementary Fig. S8a). When the level of μ or microRNAs is further decreased, high Grb2 signal can induce the cell to undergo complete EMT to attain the M phenotype, while the regulatory function of Gab1 signal depends on the level of signal μ. Gab1 induces the cells to the M phenotype at the intermediate level of μ, but to A phenotype at the low level of μ (Supplementary Fig. S8b). Therefore, the regulatory functions of Grb2 and Gab1 in inducing cell migration depend on the amount of microRNAs (miR-34 and miR-200), and their functions are more distinguished when the microRNAs are at lower levels, such that Grb2 leads to an M phenotype while Gab1 leads to an A phenotype.

**Figure 6:**
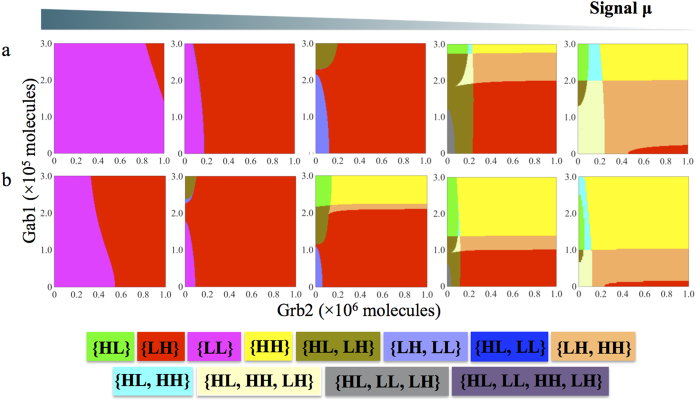
The circuit response to Grb1 and Grb2 at different levels of the μ signal. For both panels (**a,b**), the μ level for each phase diagram decreases from left to right. **(a)** For the thresholds of both the Gab1 and Grb2 signals to activate RhoA and Rac1 were set to be 500,000 molecules. At high levels of the microRNAs the 1st panel in Fig. 6a, only two phases are observed – {LL} and {LH}. As the level of μ decreases, the phases with solitary cell migration phenotypes become more common, and many multistable phases appear. **(b)** The thresholds of both Gab1 and Grb2 to activate RhoA and Rac1 were set to be 250,000 molecules. All other parameters and the μ levels for each case are same as that in Fig. 6a. The color scheme for different phases is shown at the bottom.

Next, we have calculated the two-parameter bifurcation diagrams of Rac1/RhoA circuit driven by Grb2 and Gab1 signals for various values of μ (Fig. 6a,b). For the high level of μ, the expression of Rac1 and RhoA is strongly inhibited, thus the plasticity of cell migration driven by Grb2 and Gab1 is limited only to {LL} and {LH} phases, indicating that only EMT/MET could occur (the 1st-2nd panels to the left, Fig. 6a). However, with a decrease in the levels of μ, cells can also adopt the HL state (A phenotype) (the 3rd panel to the left, Fig. 6a); and this phenotypic plasticity for cells only increases with the decrease in the signal μ. At extremely low levels of μ, the cells can adopt any of the three solitary migration phenotypes – A, M, or A/M – depending on the Gab1 and Grb2 levels (the 4th-5th panels to the left Fig. 6a). Again, these results are consistent with the experiments suggesting that the miR-200 and miR-34 inhibit cell migration and consequently metastasis, and their loss can lead the cells to display a spectrum of solitary migration phenotypes^29^,^30^. Also, we notice that this series of phase diagrams shows an asymmetry for the induction of different solitary migration phenotypes by Gab1 and Grb2 signals at different microRNA levels. Cells in the E/M phenotype may undergo a transition to the M phenotype (i.e. complete EMT) even at high levels of μ, but they can transit to the A or the A/M phenotypes only at low levels of μ. This asymmetry might underlie why different cell lines might prefer different migration phenotypes (or different distributions of migration phenotypes) during metastasis^13^.

Also, for a different parameter set where the Rac1/RhoA circuit is more responsive to Grb2 and Gab1 (the threshold for Grb2 and Gab1 signals was reduced (see supplementary information section 2)). cells can attain the {HL} phase for a larger range of parameters even in the presence of high level of μ, indicating a more metastatic behavior (the 1st-2nd panels to the left, Fig. 6b). When μ is reduced, the plasticity in cell migration is enhanced significantly (the 3rd-5th panels to the left, Fig. 6b). This difference in the response to the c-MET pathway can be attributed to different cell lines with distinct metastatic potential.

### Phenotype Distribution

**Figure 7:**
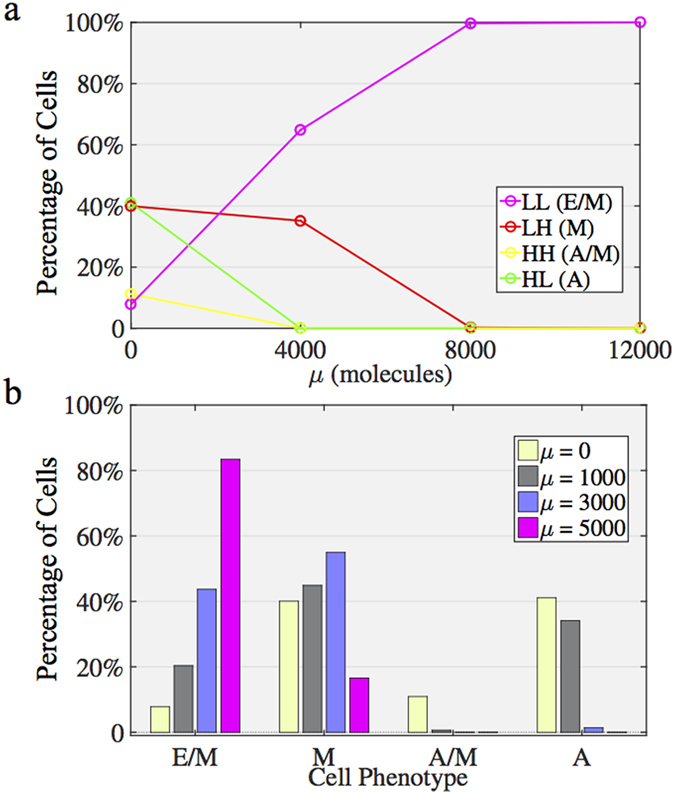
Phenotype distribution of a population of cells driven by the signal μ. (**a**) The cell parameters are randomly distributed over ±5% relative to the original parameters. The y-axis denotes the percentage of cells corresponding to a specific phenotype. The color of each line represents a phenotype. Under the different levels of μ, a population of cells has different distributions for cell phenotypes (percentage of cells shown in y-axis). (**b**) At different level of signal μ, the original phase of the cells are colored according to the color definition in Fig. 2. At high levels of the signal μ, the cells are highly likely to stay in the LL (E/M) phenotype.

Even in the same cell line, cells often have non-heritable phenotypic variability or heterogeneity^39^. Such variability can often account for different model parameters for each cell. To capture this heterogeneity, we calculated the population distribution of the phenotypes for the cells with the same levels of microRNAs, but some variability in other model parameters. More specifically, we extended our simulations to a population of 5,000 cells. Each cell has different circuit parameters, which can be ±5% different from the original parameters (details in Method section). In Fig. 7, we show the percentages of cells in one of the four different possible phenotypes—(HL, LL, LH, and HH)—for different levels of the signal μ. We found that for a high level of signal μ, a significant percentage of cells adopt the E/M phenotype (Fig. 7a,b), thereby indicating that the cells with high levels of microRNA are more robust to the parameter variability against maintaining the E/M phenotype.

## Discussion

Cellular plasticity mediated by the EMT/MET and the AMT/MAT regulatory circuits has been studied extensively individually^2^,^5^,^14^,^15^. However, understanding the regulation of these transitions together has not received enough attention. Here, we have presented, to the best of our knowledge, the first theoretical attempt to understand the interplay between EMT/MET and AMT/MAT by investigating the dynamics of the RhoA/Rac1 circuit (the core circuit for AMT/MAT) driven by miR-200 and miR-34, the gatekeepers of epithelial phenotype and inhibitors of metastasis. Our results explain how the cancer cells can attain different phenotypes and transitions among them during metastasis—collective migration as a cluster of Circulating Tumor Cells (CTCs)^40^, and different modes of individual migration—amoeboid (A), mesenchymal (M), or hybrid A/M phenotype^5^,^7^,^11^,^12^,^13^. We further present several predictions about how microRNAs and stromal signals such as HGF affect these transitions.

Our previous work showed that the RhoA/Rac1 circuit could perform as a three-way switch allowing three stable steady states – (high RhoA-GTP, low Rac1-GTP), (low RhoA-GTP, high Rac1-GTP), and (high RhoA-GTP, high Rac1-GTP). These three states can be associated with the A, the M and the hybrid A/M phenotypes respectively^14^, as supported by several experimental evidences. For example, amoeboid cells have been shown to have high actomyosin contractility due to high levels of RhoA-GTP^41^; mesenchymal cells have high actin polymerization due to high levels of Rac1-GTP^41^; the hybrid A/M phenotype displays mixed amoeboid and mesenchymal characteristics, suggesting a comparable level of both actin polymerization and actomyosin contractility due to high levels of both Rac1-GTP and RhoA-GTP^7^,^11^,^12^,^13^. Notably, similar ‘hybrid’ manifestations of mixed amoeboid and mesenchymal traits may not be unique to cancer, but can also be observed in other instances, such as neutrophils and leukocytes^42^,^43^,^44^.

When incorporating the microRNA signaling on the RhoA/Rac1 circuit, we observed an additional stable state (LL), when cells of which have relatively low levels of both RhoA-GTP and Rac1-GTP as compared to the other states (LH, HH, and HL). We propose to associate this new state with the hybrid E/M phenotype. Although there is no direct quantitative measurement yet of the active levels of RhoA-GTP and Rac1-GTP in the hybrid E/M cells, our association of the LL state with hybrid E/M phenotype is consistent with the following experiments. First, moderate levels of both active Rac1 and RhoA have been reported to promote wound healing^27^, a typical case of the collective migration of the hybrid E/M cells^2^. Second, a moderate level of active RhoA has been shown to induce the hybrid E/M phenotype for the human colon adenocarcinoma cell^26^, while both dominant-negative and constitutively active Rac1 has been reported to damage the collective migration of boarder cells during Drosophila early development^28^. Both evidences support the relatively low activity of Rac1 and RhoA in collectively migrating hybrid E/M cells as compared with the solitarily migrating cells in A, M and A/M phenotypes.

One limitation of our current model is the exclusion of the epithelial (E) phenotype from our analysis because this model is too simple to explain the elusive dynamics of active RhoA and Rac1 in epithelial tissue establishment and maintenance. Epithelial cells are reported to have higher levels of microRNAs (miR-200 and miR-34) than in the hybrid E/M cells^15^,^45^. Therefore, according to our model, these cells are likely to have even lower levels of active RhoA and Rac1 than those in hybrid E/M cells. On the other hand, experiments suggest that the active levels of RhoA and Rac1 vary significantly in different stages of epithelial establishment^46^. For instance, at the initial stage of epithelialization, *de novo* cell-cell adhesion and adherens junctions expansion require the activity of Rac1 and RhoA respectively. But when EMT is induced, levels of active Rac1 decrease at first and then increase again^47^. Therefore, the dynamics of Rac1/RhoA activity need to be fine-tuned either spatially and/or temporally in order to maintain the E-cadherin-mediated cell-cell adhesion. But we currently have not yet modeled the spatial heterogeneity of the two GTPases, RhoA and Rac1, as needed to allow investigation of the role of spatiotemporal dynamics of these proteins in determining the changes in cell shape during epithelialization and transitions between different phenotypes. Besides, to understand the complex temporal dynamics of the activity of Rac1 and RhoA, additional models should incorporate the interplay among the key proteins in the regulatory circuit such as E-cadherin, N-cadherin, and the Rho GTPases.

From the perspective of dynamical systems, we found that the Rac1/RhoA regulatory circuit can behave as a multi-stable switch in the regulation of different phenotypes. Such multi-stability is a hallmark of self-activating toggle switches (SATS), i.e. mutually inhibitory feedback loop with self-activations on both elements of the loop^48^ as seen in RhoA/Rac1 circuit (Fig. 1b). Similar multi-stability is also seen in circuits governing Cancer Stem Cells (CSCs)^49^,^50^ and in circuits governing cell-cell communication^51^,^52^, and can often be used by the cancer cells to adapt to their rapidly changing microenvironments during metastasis^4^. Restricting such cellular plasticity as enabled by these multistable systems can inhibit possible co-operation between different subpopulations and hence hamper tumor metastasis^53^,^54^,^55^,^56^,^57^.

To conclude, we present the first tractable framework towards understanding the transitions among the collective migration phenotype and the solitary migration phenotypes. We found that the microRNAs, miR-34 and miR-200, govern various phenotypic transitions and can also mediate the effect of other signaling pathways such as Grb2/Gab1 signals on such transitions. Our framework can be further extended by incorporating many other extracellular signals to explore their impact on cellular plasticity and migration, and can possibly provide important insights into cancer therapies that target cell migration during metastasis.

## Method

### Deterministic analysis

The dynamics of the coupled circuit is described by a set of six nonlinear chemical rate equations as shown in equation (S3). However, this model can be further simplified into two rate equations shown in equation 1 (Details in Supplementary Information section 1). Thereafter, we performed stability analysis on the effective model. To compute all the possible steady state solutions, we calculated two nullclines – the first one satisfies 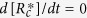 (solid red line, Fig. 1c), and the second one satisfies 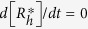 (solid black line, Fig. 1c). The nullclines are constructed on the phase plane by the concentrations of RhoA-GTP (x-axis) and Rac1-GTP (y-axis). The intersections of these two lines are the steady states for the whole system. The stability of the steady states can be further determined by the linear approximation method^58^. The nullclines are computed by the contour-based method (contour() function in Matlab), and the bifurcation diagrams are calculated by the MatCont package^59^. Besides, we simulated the temporal dynamics of the circuit (described by equation (S3)) by using the ode15s solver in Matlab.

Similar to our previous work, we modeled the Grb2 and Gab1 signals 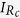 and 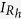 as follows:


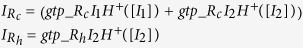


The model parameters are listed in Supplementary Information section 2.

### Stochastic simulation

Here, we started from equation (S3), which can be symbolized by 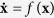, where 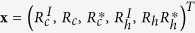. Since the binding and unbinding of Rho GTPases with GDP-dissociation inhibitor (GDI) is fast (see Supplementary Information section 2), we considered these process to attain equilibrium rapidly, thus equation (S3) can be reduced to four rate equations with the rate equations for Rac1-GDI and Rho-GDI set to 0. In this study, we assumed the dynamics of the circuit to be influenced by a Gaussian white noise. So the dynamics can be described by a Langenvin equation 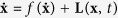, where 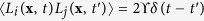 for 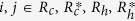, ϒ is a constant representing the noise level. The stochastic differential equation is integrated by the Euler-Maruyama method^60^. The effective landscape for the state **X** is defined by *E* = −ln(*P*(**x**))^31^,^32^, where *P*(**x**) is the probability for the system in the state x. The Langevin simulation gives a long time trajectory (10^6^ hour) of the protein expressions of the circuit, from which we computed the probability of being in any of the steady states, and the rates of the transitions among these states (details in Supplementary Information section 7).

### Phenotype distribution

To mimic the heterogeneity of a cell population, we randomly generated 5,000 sets of circuit parameters, in which each parameter was randomly perturbed away from the original value by at most 5% (random sampling follows a uniform distribution). For each of the Hill coefficients, it follows a discrete uniform distribution within [n − 1, n + 1], where n is the original value. We evaluated the dynamics of the circuit for each cell by modeling the circuit with each randomized set of parameters. We performed stability analysis, and calculated all stable steady states, each of which was mapped to the corresponding phenotypes based on the levels of the proteins. The mapped phenotypes were weighted by the number of stable steady states for each cell to calculate the fraction of cells in each phenotype.

The relevant Matlab and C codes are accessible at https://rice.app.box.com/s/7vfm90cwh4lstnpsa72xhlairugdj6hh.

## Additional Information

**How to cite this article**: Huang, B. *et al.* Modeling the Transitions between Collective and Solitary Migration Phenotypes in Cancer Metastasis. *Sci. Rep.* **5**, 17379; doi: 10.1038/srep17379 (2015).

## Supplementary Material

Supplementary Information
