# Supplementary material for: Modeling the Transitions between Collective and Solitary Migration Phenotypes in Cancer Metastasis
Source: Sci Rep. 2015 Dec 2;5:17379. doi: 10.1038/srep17379 (PMC4667179; doi:10.1038/srep17379)
Supplement: Supplementary Information [file srep17379-s1.pdf]

Modeling the Transitions between Collective and Solitary Migration Phenotypes in Cancer Metastasis

Bin Huang[1,2], Mohit Kumar Jolly [1,3], Mingyang Lu[1], Ilan Tsarfaty[5,*],
Eshel Ben-Jacob [1,6,7], Jose' N Onuchic [1,2,4,6,*]

[1]Center for Theoretical Biological Physics, [2]Department of Chemistry, [3]Department of Bioengineering, [4]Department of Physics and Astronomy, [6]Department of Biosciences, Rice University, Houston, TX 77005-1827, USA
[5] Department of Clinical Microbiology and Immunology, Sackler School of Medicine
[7]School of Physics and Astronomy and The Sagol School of Neuroscience,
Tel-Aviv University, Tel-Aviv 69978, Israel

[*]Authors to whom correspondence should be sent: Ilan Tsarfaty (Email: ilants@post.tau.ac.il); Jose' N Onuchic (Email: jonuchic@rice.edu).

Supplementary Information

1. Details about Mathematical Model for the Coupled Regulatory Circuit

Rac1 and RhoA are belonged to the Rho family of small GTPases, which could act as molecular switches among their active (the GTP-bound state) and inactive (the GDP-bound state and the GDI-bound state) forms[1]. Three sets of proteins involve in the regulations of these switches: Guanine nucleotide exchange factors (GEFs) that elevate the levels of the active GTPases by exchanging the bound GDP with GTP; GTPase-activating proteins (GAPs) that reduce the concentration of the active GTPases by promoting the GTP hydrolysis rates; and guanine nucleotide dissociation inhibitors (GDIs) that inactivate the GTPases by sequestering them from the membrane to the cytosol[1]. In our previous work, we developed a theoretical framework for Rac1/RhoA GTPaeses-based circuit[2], as shown in equation (S1).

$$\frac{d[R_c^I]}{dt} = gdi\_R_c \bullet [R_c] - dgdi\_R_c \bullet [R_c^I]$$

$$\frac{d[R_c]}{dt} = g_{R_c} + J_{R_c^*}([R_h^*]) \bullet [R_c^*] + dgdi\_R_c \bullet [R_c^I]$$
$$- gdi\_R_c \bullet [R_c] - B_{R_c}([R_c^*]) \bullet [R_c] - K_{R_c} \bullet [R_c]$$

$$\frac{d[R_c^*]}{dt} = B_{R_c}([R_c^*]) \bullet [R_c] - J_{R_c^*}([R_h^*]) \bullet [R_c^*] - K_{R_c^*} \bullet [R_c^*]$$

$$\frac{d[R_h^I]}{dt} = gdi\_R_h \bullet [R_h] - dgdi\_R_h \bullet [R_h^I]$$

$$\frac{d[R_h]}{dt} = (g_{R_h} + g_{R_hA} \bullet H_{R_h}^+([R_h^*])) + J_{R_h^*}([R_c^*]) \bullet [R_h^*] + dgdi\_R_h \bullet [R_h^I]$$
$$- gdi\_R_h \bullet [R_h] - B_{R_h}([R_h^*]) \bullet [R_h] - K_{R_h} \bullet [R_h]$$

$$\frac{d[R_h^*]}{dt} = B_{R_h}([R_h^*]) \bullet [R_h] - J_{R_h^*}([R_c^*]) \bullet [R_h^*] - K_{R_h^*} \bullet [R_h^*] \quad \text{, (S1)}$$

where

$$B_{R_c}([R_c^*]) = gtp\_R_ci + gtp\_R_cB + gtp\_R_cA \bullet H_{R_c}^+([R_c^*])$$
$$J_{R_c^*}([R_h^*]) = dgtp\_R_ci + dgtp\_R_cB + dgtp\_R_cA \bullet H_{R_c^*}^+([R_h^*])$$

$$B_{R_h}([R_h^*]) = gtp\_R_hi + gtp\_R_hB + gtp\_R_hA \bullet H_{R_h}^+([R_h^*])$$
$$J_{R_h^*}([R_c^*]) = dgtp\_R_hi + dgtp\_R_hB + dgtp\_R_hA \bullet H_{R_h^*}^+([R_c^*])$$

Here, R_c^I represents the GDI-bound state of Rac1, R_c represents the GDP-bound state of Rac1, and R_c^* represents the GTP-bound state of Rac1. Same notations hold for RhoA (R_h^I, R_h, R_h^*). g_{R_c} and g_{R_h} stand for the basal production rate of Rac1 and RhoA, respectively, and g_{R_hA} are the excitatory production rate of RhoA resulting from its transcriptional self-activation. $K_{R_c^*}$ and $K_{R_h^*}$ are the corresponding degradation rates for Rac1 and RhoA. B function is the total GTP loading rate constant. It contains the intrinsic GTP loading rate constant (gtp_R_ci and gtp_R_hi), the activated GTP loading rate constant (gtp_R_cB and gtp_R_hB) by GEFs not involved in the auto-regulations and the activated GTP loading rate constant (gtp_R_cA and gtp_R_hA) resulting from auto-

regulations. J function is the total GTP hydrolysis rate constant, which also contains three parts: the intrinsic GTP hydrolysis rate constant ($dgtp\_R_c i$ and $dgtp\_R_h i$), the activated GTP hydrolysis rate constant ($dgtp\_R_c B$ and $dgtp\_R_h B$) by GAPs not involved in the mutually regulations and the activated GTP hydrolysis rate constant ($dgtp\_R_c A$ and $dgtp\_R_h A$) resulting from the mutual regulations. The Hill function $H_y^+([x])$ represents the regulation from protein x to protein y, for example $H_{R_h}^+([R_h^*])$ in equation (S1) stands for the transcriptional self-activation of RhoA. $H_y^+([x])$ is defined as $(x/x_0)^{n_x}/(1+(x/x_0)^{n_x})$, where x is the level of regulator x, x_0 and n_x stand respectively for the threshold and the co-operativity for the regulation from protein x to protein y. gdi_R_c, $dgdi\_R_c$, gdi_R_h, and $dgdi\_R_h$ are the binding and unbinding rate constants for GDI.

EMT regulatory circuit is consist of two coupled toggle switches: ZEB/miR-200 and SNAIL/miR-34[3]. It can inhibit Rac1/RhoA circuit via miR-200 and miR-34[4–8]. Both of them could inhibit the translation of either Rac1 or RhoA. Here, we adopt the theoretical framework for microRNA-based circuit from our previous work to incorporate the roles of microRNAs in regulating Rac1/RhoA circuit as follows[9]:

$$
\begin{aligned}
&\frac{d[mR_c]}{dt} = g_{mR_c} - [mR_c]\bullet Y_{mR_c}(\mu_2) - K_{mR_c}\bullet[mR_c] \\
&\frac{d[R_c{}^I]}{dt} = gdi\_R_c \bullet [R_c] - dgdi\_R_c \bullet [R_c{}^I] \\
&\frac{d[R_c]}{dt} = g_{PR_c}\bullet[mR_c]\bullet L_{R_c}(\mu_2) + J_{R_c^*}([R_h^*])\bullet[R_c^*] + dgdi\_R_c\bullet[R_c{}^I] \\
&\qquad\qquad - gdi\_R_c\bullet[R_c] - B_{R_c}([R_c^*])\bullet[R_c] - K_{R_c}\bullet[R_c] \\
&\frac{d[R_c^*]}{dt} = B_{R_c}([R_c^*])\bullet[R_c] - J_{R_c^*}([R_h^*])\bullet[R_c^*] - K_{R_c^*}\bullet[R_c^*]
\end{aligned}
$$

$$
\begin{aligned}
&\frac{d[mR_h]}{dt} = (g_{mR_h} + g_{mR_hA}\bullet H^+_{R_h^*}([R_h^*])) - [mR_h]\bullet Y_{mR_h}(\mu_1) - K_{mR_h}\bullet[mR_h] \\
&\frac{d[R_h{}^I]}{dt} = gdi\_R_h \bullet [R_h] - dgdi\_R_h \bullet [R_h{}^I] \\
&\frac{d[R_h]}{dt} = g_{PR_h}\bullet[mR_h]\bullet L_{R_h}(\mu_1) + J_{R_h^*}([R_c^*])\bullet[R_h^*] + dgdi\_R_h\bullet[R_h{}^I] \\
&\qquad\qquad - gdi\_R_h\bullet[R_h] - B_{R_h}([R_h^*])\bullet[R_h] - K_{R_h}\bullet[R_h] \\
&\frac{d[R_h^*]}{dt} = B_{R_h}([R_h^*])\bullet[R_h] - J_{R_h^*}([R_c^*])\bullet[R_h^*] - K_{R_h^*}\bullet[R_h^*]
\end{aligned}
\quad , \text{(S2)}
$$

where

$$
\begin{aligned}
&Y_m(\mu) = \sum_{i=1}^{n} \gamma_{mi} C_n^i M_n^i(\mu); \\
&L(\mu) = \sum_{i=0}^{n} l_i C_n^i M_n^i(\mu); \\
&M_n^i(\mu) = (\mu/\mu_0)^i / (1+\mu/\mu_0)^n;
\end{aligned}
$$

mR_c is the mRNA of Rac1 and mR_h is the one for RhoA. g_{mR_c} and g_{mR_h} are the transcriptional rates for Rac1 and RhoA, respectively, while K_{mR_c} and K_{mR_h} are degradation rates for them, respectively. g_{mR_hA} is the excitatory transcriptional rate due to the transcriptional self-activation of RhoA protein. g_{R_c} and g_{R_h} are the translation rates of protein Rac1 and RhoA respectively. Y function describes the microRNA (μ)-dependent degradation of mRNAs, and L function represents the inhibited translations by microRNAs. μ_0 is the threshold for P function and n is the number of binding sites a

microRNA has on a given mRNA[9]. μ_1 and μ_2 are the microRNAs regulating RhoA and Rac1 respectively.

Here, we assumed that quasi-equilibrium for the transcription process of protein Rac1 and RhoA ($d[mR_c]/dt = 0; d[mR_h]/dt = 0$) because the innate degradation rate of mRNA is about five to ten times faster than that of proteins[10]. Equation (S2) can be simplified as follow:

$$
\begin{aligned}
\frac{d[R_c^{\,I}]}{dt} &= gdi\_R_c \bullet [R_c] - dgdi\_R_c \bullet [R_c^{\,I}] \\
\frac{d[R_c]}{dt} &= g_{R_c} \bullet P_{R_c}([\mu_2]) + J_{R_c^*}([R_h^*]) \bullet [R_c^*] + dgdi\_R_c \bullet [R_c^{\,I}] \\
&\quad - gdi\_R_c \bullet [R_c] - B_{R_c}([R_c^*]) \bullet [R_c] - I_{R_c} \bullet [R_c] - K_{R_c} \bullet [R_c] \\
\frac{d[R_c^*]}{dt} &= B_{R_c}([R_c^*]) \bullet [R_c] + I_{R_c^*} \bullet [R_c] - J_{R_c}([R_h^*]) \bullet [R_c^*] - K_{R_c^*} \bullet [R_c^*]
\end{aligned} \quad , \text{(S3)}
$$

$$
\begin{aligned}
\frac{d[R_h^{\,I}]}{dt} &= gdi\_R_h \bullet [R_h] - dgdi\_R_h \bullet [R_h^{\,I}] \\
\frac{d[R_h]}{dt} &= (g_{R_h} + g_{R_hA} \bullet H^+_{R_h^*}([R_h^*])) \bullet P_{R_h}([\mu_1]) + J_{R_h^*}([R_c^*]) \bullet [R_h^*] + dgdi\_R_h \bullet [R_h^{\,I}] \\
&\quad - gdi\_R_h \bullet [R_h] - B_{R_h}([R_h^*]) \bullet [R_h] - I_{R_h} \bullet [R_h] - K_{R_h} \bullet [R_h] \\
\frac{d[R_h^*]}{dt} &= B_{R_h}([R_h^*]) \bullet [R_h] + I_{R_h} \bullet [R_h] - J_{R_h^*}([R_c^*]) \bullet [R_h^*] - K_{R_h^*} \bullet [R_h^*]
\end{aligned}
$$

where

$$
\begin{aligned}
&P_X([\mu]) = L_X([\mu]) / (1 + Y_{mX}([\mu]) / k_{mX}) \\
&g_{R_c} = g_{mR_c} g_{PR_c} / K_{mR_c}; \\
&g_{R_h} = g_{mR_h} g_{PR_h} / K_{mR_h}; \\
&g_{R_hA} = g_{mR_hA} g_{PR_h} / K_{mR_h};
\end{aligned}
$$

Furthermore, assuming the total expression of Rac1 and RhoA reach equilibrium, therefore

$$\frac{d[R_{ctot}]}{dt} = \frac{d[R_c^I]}{dt} + \frac{d[R_c]}{dt} + \frac{d[R_c^*]}{dt} = 0$$
$$\frac{d[R_{htot}]}{dt} = \frac{d[R_h^I]}{dt} + \frac{d[R_h]}{dt} + \frac{d[R_h^*]}{dt} = 0 \quad , \text{(S4)}$$

Then, we can further reduce the model to two coupled equations for active Rac1 and RhoA:

$$\begin{aligned}\frac{d[R_c^*]}{dt} &= \frac{g_{R_c}}{K_{R_c^*}} P_{R_c}([\mu_2])(B_{R_c}([R_c^*]) + I_{R_c}) \\ &\quad - (B_{R_c}([R_c^*]) + J_{R_c^*}([R_h^*]) + I_{R_c} + K_{R_c^*})[R_c^*] \\ \frac{d[R_h^*]}{dt} &= \frac{(g_{R_h} + g_{R_h A} H^+_{R_h^*}([R_h^*]))}{K_{R_h^*}} P_{R_h}([\mu_1])(B_{R_h}([R_h^*]) + I_{R_h}) \\ &\quad - (B_{R_h}([R_h^*]) + J_{R_h^*}([R_c^*]) + I_{R_h} + K_{R_h^*})[R_h^*]\end{aligned} \quad , \text{(S5)}$$

2. Parameters Estimation

miR-34a could either suppress the transcription of RhoA by targeting its transcriptional complexes or directly Rac1[4,5]. Also, the bioinformatics tool, TargetScan[11–14], indicates the possible inhibition of miR-200 on Rac1 and RhoA, which is consistent with several experimental results[6–8]. To simplify these connections between miR-34/miR-200 and Rac1/RhoA, we here consider both of them directly inhibit the expression of Rac1 and RhoA, and group their inhibitions on RhoA to be ' μ_1 ' while the ones on Rac1 to be ' μ_2 '. The effective binding sites for μ_1 and μ_2 on both RhoA and Rac1 is set to 2, and we use the same parameters for the P functions as we used before[3], as shown in Fig. S1. Therefore, the protein expressions of target proteins of these microRNAs come down to 30% of the control case (no inhibition by microRNAs). The thresholds for P function is set to be 10, 000 molecules, but it should be noted that it is the ratio between the concentration of microRNAs to their threshold levels instead of their absolute concentration values that actually governs the effects of these signals. For the part of Rac1/RhoA circuit, we continue to use the same parameters from our previous work for Rac1/RhoA regulatory circuit[2].

Supplementary Table S2. Parameters Table

Parameters	Value	Unit	Description
Rac1			
g_{R_c}	3.4×10^5	molecules/h	Production rate
K_{R_c}	0.1	h^{-1}	Degradation rate for Rac1-GDP
$K_{R_c^*}$	0.1	h^{-1}	Degradation rate for Rac1-GTP
gdi_R_c	2.0×10^3	h^{-1}	Binding rate for GDI to Rac1-GDP
$dgdi\_R_c$	2.0×10^3	h^{-1}	Dissociation rate for Rac1-GDI
$gtp\_R_c i$	0.54	h^{-1}	Intrinsic GTP loading rate
$gtp\_R_c B$	19.46	h^{-1}	Activated GTP loading rate
$gtp\_R_c A$	530	h^{-1}	Activated GTP loading rate by self-activation
$gtp\_R_c A_0$	1.9×10^6	molecules	Threshold for self-activation
$n_{gtp\_R_c A}$	4	-	Hill coefficient for self-activation
$dgtp\_R_c i$	6.6	h^{-1}	Intrinsic GTP hydrolysis rate
$dgtp\_R_c B$	105.4	h^{-1}	Activated GTP hydrolysis rate
$dgtp\_R_c A$	198	h^{-1}	Activated GTP hydrolysis rate by RhoA
$dgtp\_R_c A_0$	1.2×10^6	molecules	Threshold for RhoA inactivation
$n_{dgtp\_R_c A}$	4	-	Hill coefficient for RhoA inactivation
RhoA			
g_{R_h}	1.6×10^5	molecules/h	Basal production rate
$g_{R_h A}$	3.4×10^5	molecules/h	Excitatory production rate
$g_{R_h A_0}$	8.0×10^5	molecules	Threshold for transcriptionally self-activation
$n_{g_{R_h A}}$	4	-	Hill coefficient for self-activation
K_{R_h}	0.1	h^{-1}	Degradation rate for RhoA-GDP
$K_{R_h^*}$	0.1	h^{-1}	Degradation rate for RhoA-GTP
gdi_R_h	2.0×10^3	h^{-1}	Binding rate for GDI to RhoA-GDP
$dgdi\_R_h$	2.0×10^3	h^{-1}	Dissociation rate for RhoA-GDI

$gtp\_R_h i$	0.54	h^{-1}	Intrinsic GTP loading rate
$gtp\_R_h B$	109.46	h^{-1}	Activated GTP loading rate
$gtp\_R_h A$	196	h^{-1}	Activated GTP loading rate by self-activation
$gtp\_R_h A_0$	1.0×10^6	molecules	Threshold for self-activation
$n_{gtp\_R_h A}$	4	-	Hill coefficient for self-activation
$dgtp\_R_h i$	1.32	h^{-1}	Intrinsic GTP hydrolysis rate
$dgtp\_R_h B$	308.68	h^{-1}	Activated GTP hydrolysis rate
$dgtp\_R_h A$	89	h^{-1}	Activated GTP hydrolysis rate by Rac1
$dgtp\_R_h A_0$	1.3×10^6	molecules	Threshold for Rac1 inactivation
$n_{dgtp\_R_h A}$	4	-	Hill coefficient for Rac1 inactivation
c-Met Signals			
$gtp\_R_c I_1$	240	h^{-1}	Activated GTP loading rate for Rac1 by Grb2
$gtp\_R_c I_{10}{}^*$	5.0×10^5	molecules	Threshold for Grb2 activation on Rac1
$n_{gtp\_R_c I_1}$	2	-	Hill coefficient for Grb2 activation on Rac1
$gtp\_R_h I_2$	240	h^{-1}	Activated GTP loading rate for RhoA by Gab1
$gtp\_R_h I_{20}{}^*$	5.0×10^5	molecules	Threshold for Gab1 activation on RhoA
$n_{gtp\_R_h I_2}$	2	-	Hill coefficient for Gab1 activation on RhoA
$gtp\_R_c I_2$	90	h^{-1}	Activated GTP loading rate for Rac1 by Gab1
$gtp\_R_c I_{20}$	5.0×10^5	molecules	Threshold for Gab1 activation on Rac1
$n_{gtp\_R_c I_2}$	2	-	Hill coefficient for Gab1 activation on Rac1
microRNAs			
n_μ	2	-	Hill coefficient for microRNAs
l_0	1	-	Coefficient for $L(\mu)$
l_1	0.6	-	Coefficient for $L(\mu)$
l_2	0.3	-	Coefficient for $L(\mu)$
γ_{m1}	0.04	h^{-1}	Coefficient for $Y_m(\mu)$
γ_{m2}	0.2	h^{-1}	Coefficient for $Y_m(\mu)$

K_{mR_c}	0.5	h^{-1}	Degradation rate for mRNA of Rac1
K_{mR_c}	0.5	h^{-1}	Degradation rate for mRNA of RhoA
μ_0	1.0×10^4	molecules	Threshold for microRNA regulation

* There two thresholds for Grb2 and Gab1 regulations are changed to 2.5×10^5 molecules for Fig. 6b.

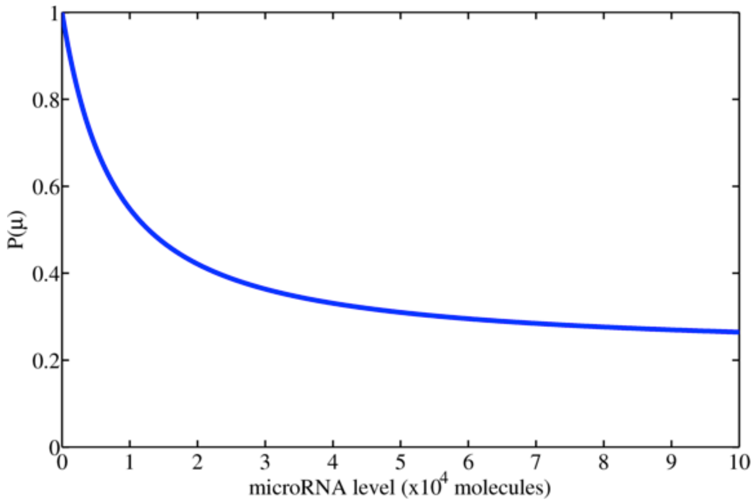

Supplementary Figure S1. Behavior of P function. P function decreases with the increase of microRNA level (μ). When there are two binding sites and the threshold is set to 10,000 molecules, the minimal value of P function is about 0.3, which means the maximum inhibition effect on a protein is about 30% of its initial levels.

3. Quadri-stable Steady State System.

In rare parameter ranges, the coupled circuit could be a quadri-stable system, where the four states (LL, LH, HH, HL) could co-exist. However, the states for LL and HL are very close to the unstable states that are represented by hollow green dots in Fig. S2. This indicates the low stability of these states in this condition.

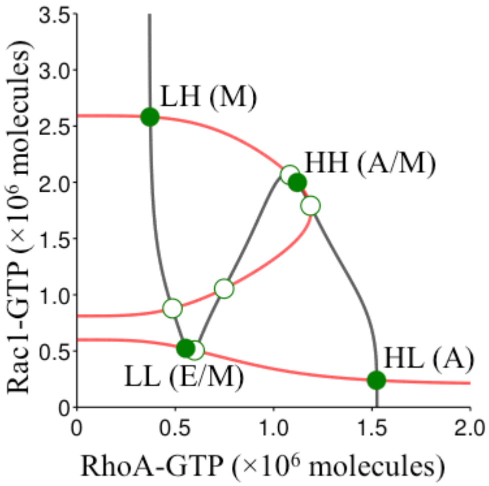

Supplementary Figure S2. Quadri-stability of the circuit. The plot shows the nullclines and possible steady states when μ_1 = 101 molecules and μ_2 = 430 molecules. The circuit is quadri-stable. Red nullcline is for $d[R_c^*]/dt$ and black nullcline is for $d[R_h^*]/dt$. Green solid circles denote the stable fixed points, and green hollow circles denote the unstable fixed points. Each stable point can be associated with a cell phenotype labeled beside it.

4. RhoA/Rac1 Circuit Response to microRNA Signals.

Here, we show the one-parameter bifurcation diagram for the circuit response to microRNA signal (μ), where μ_1 and μ_2 are treated to be equal at all times and change simultaneously. The diagram is similar to Fig. 2 but it is in the term of the protein level of active RhoA.

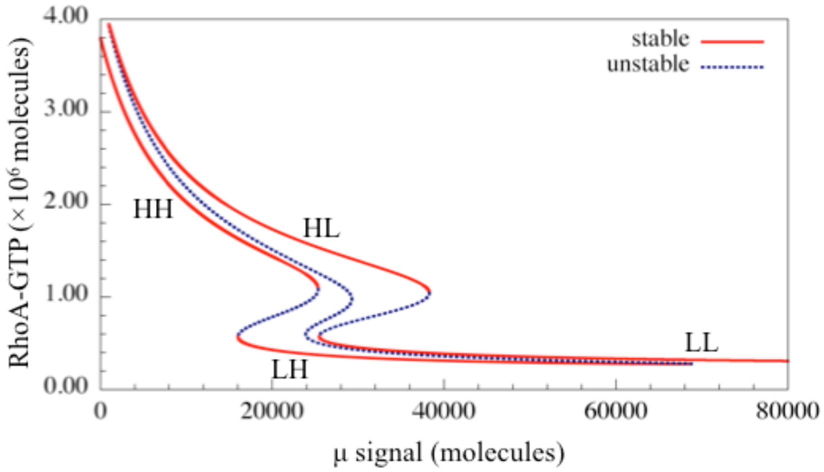

Supplementary Figure S3. Bifurcation of RhoA-GTP protein levels in response to signal μ. This diagram is complementary to Fig. 2, but it is in the term of RhoA-GTP protein level. The red solid lines indicate stable states and the blue dashed lines indicate unstable states.

5. Nullclines for different phases in two-parameter Bifurcation Diagram for μ_1 and μ_2.

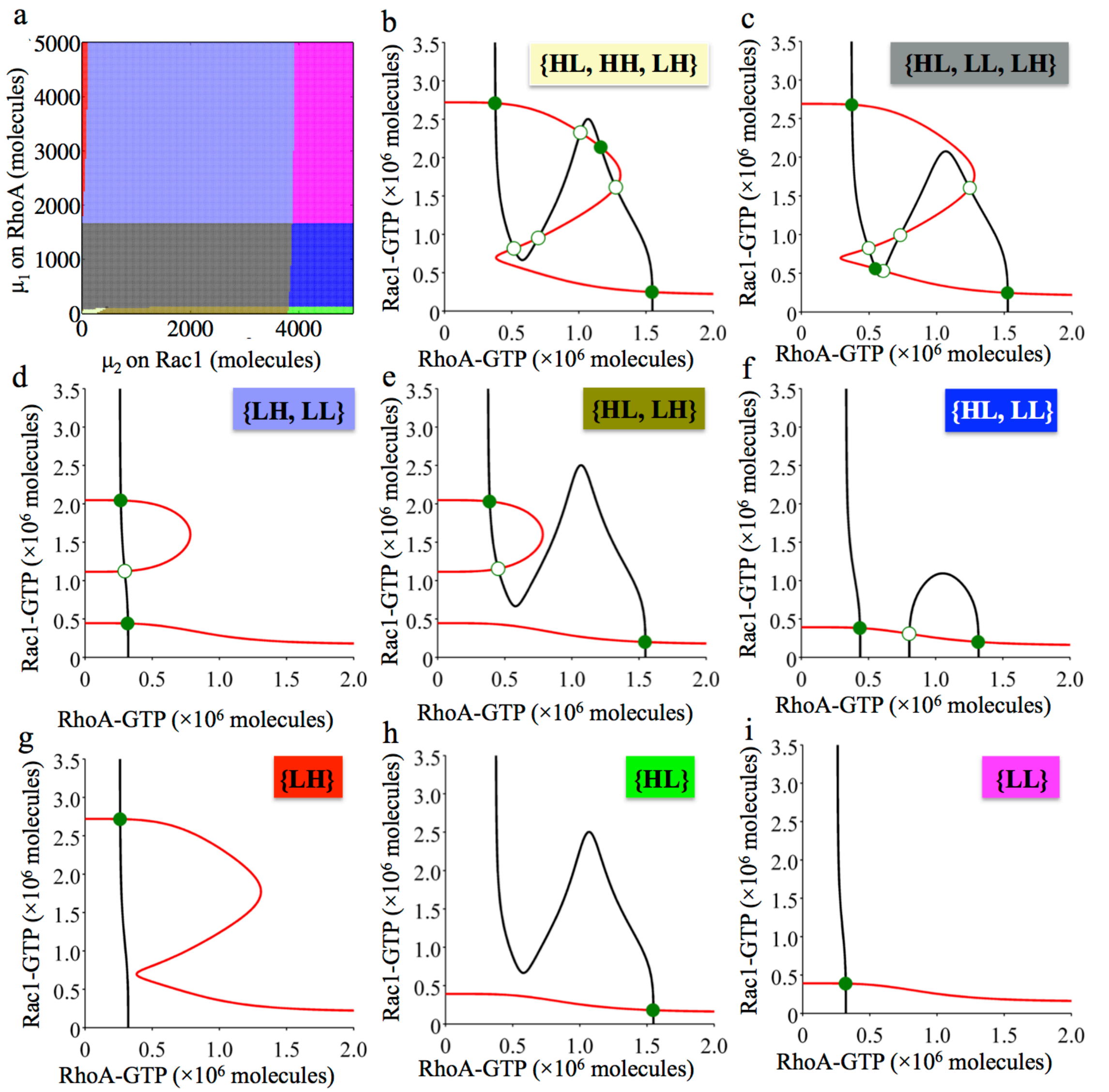

Supplementary Figure S4. Different phases in μ_1 / μ_2 phase diagram for Rac1/RhoA regulatory circuit. **(a)** Phase diagram using μ_1 (y-axis) and μ_2 (x-axis) signals as two parameters (also in Fig. 3a in the main text). **(b)** to **(h)** show the nullclines for the circuit at different phases - two phase for tri-stability **(b, c)**, three phases for bi-stability **(d, e, f)** and three phases for mono-stability **(g, h, i)**.

6. Two-Parameter Bifurcation Diagrams with Different Inhibition Strength of microRNAs.

In current parameters, the inhibition strength on RhoA and Rac1 by μ_1 and μ_2 are equal (Fig. S5a). To explore the cases with different strength, we either enhance the inhibition of μ_1 (Fig. S5b) or the one of μ_2 (Fig. S5c) by modifying the threshold for P function. As shown below, stronger inhibitions on RhoA by μ_1 make the area for the phases ({LH}, {LH, LL}, {LL}) containing LH (M phenotype) and LL (E/M phenotype) broader while stronger inhibitions on Rac1 by μ_2 make the area for the phases ({HL}, {HL, LL}, {LL}) containing HL (A phenotype) and LL (E/M phenotype) broader.

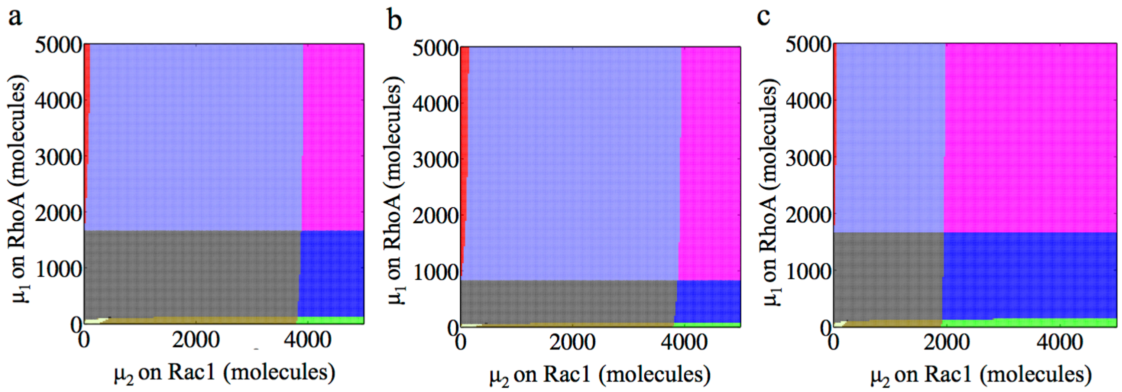

Supplementary Figure S5. Phase diagrams for different strength of microRNA inhibition. (a) The current model, whose threshold for P function for either μ_1 or μ_2 is 10,000 molecules. (b) The threshold for P function of μ_1 is 5,000 molecules, and other parameters are same as current model. (c) The threshold for P function of μ_2 is 5,000 molecules, and other parameters are same as current model.

7. Stochastic Transitions among different states

Here, in order to investigate the transitions among different states, we selected several boundaries to define each state (different color area shown in Fig. S6). As shown in Fig. S6, only the transition from one area to another different area such as the trajectory 1 is treated as being a successful transition. Thus, the trajectory 2 is not counted as a transition.

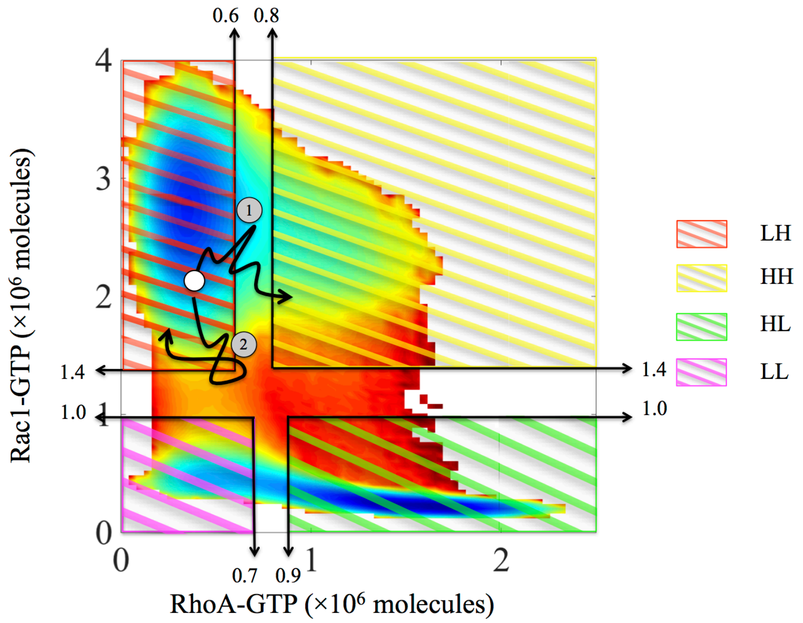

Supplementary Figure S6. Boundaries for each state. Four different color areas are defined to distinguish the four possible states (LL, LH, HL, HH). The numbers by the arrow indicate the position of the boundaries. Trajectory 1 and 2 both started from same initial state, but trajectory 1 finally make successful transition from LH to HH while trajectory 2 failed and returned to LH state.

Based on these boundaries, we could calculate the number of transition per hour for specific transition (Fig. S7a) and the probability of specific state (Fig. S7b). The

probability of specific state is defined as the ratio of accumulating time staying in the state over the total time of the simulation (10^6 hours). More transitions occur between LH and LL or HL and LL. The other transitions are rare in the present of Gaussian Noise. However, when microRNAs levels are high, all the transitions are blocked. Also, as shown in Fig. S7, when microRNAs level increase, the probability for LH and HL state decrease but that for LL state increases. Thus, at high level of signal μ, cells stay in LL state and it's hard for them to have transitions to other states.

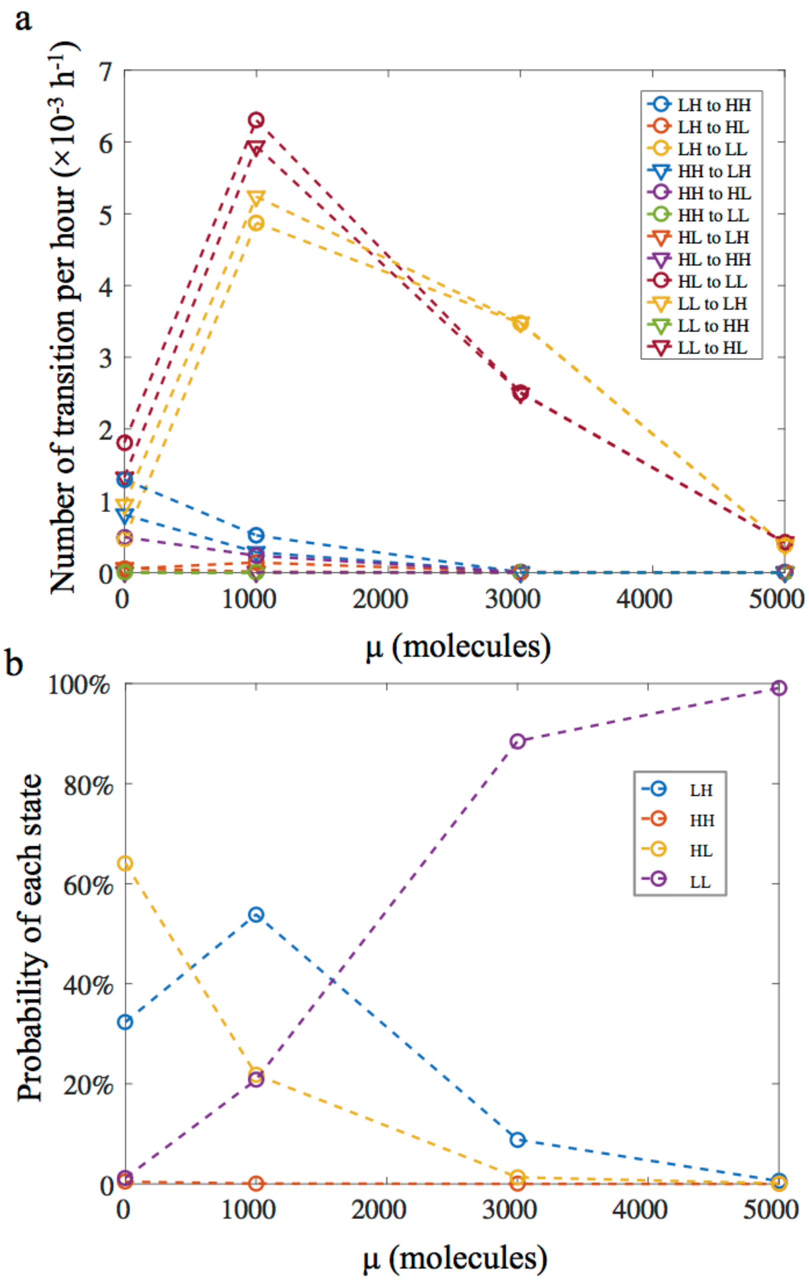

Supplementary Figure S7. Number of transition per hour between different states and the probability of each state. (a) At different level of signal μ (0, 1000, 3000, and 5000 molecules), the number of transition per hour for 12 possible transitions are shown by different lines. (b) At different level of signal μ, the probability of each state (LL, LH, HH, HL) is shown by different lines.

8. Response to input signals from c-Met pathway

Here, we show two-parameter bifurcation diagrams for μ/Grb2 (Fig. S8a) and μ/Gab1 (Fig. S8b). High Grb2 signal can induce the cell to undergo complete EMT to attain an M phenotype when microRNA is low, while the regulatory function of Gab1 signal depends on the level of signal μ. It induces the cells to an M phenotype when signal μ is at the intermediate level but to A phenotype when signal μ is low.

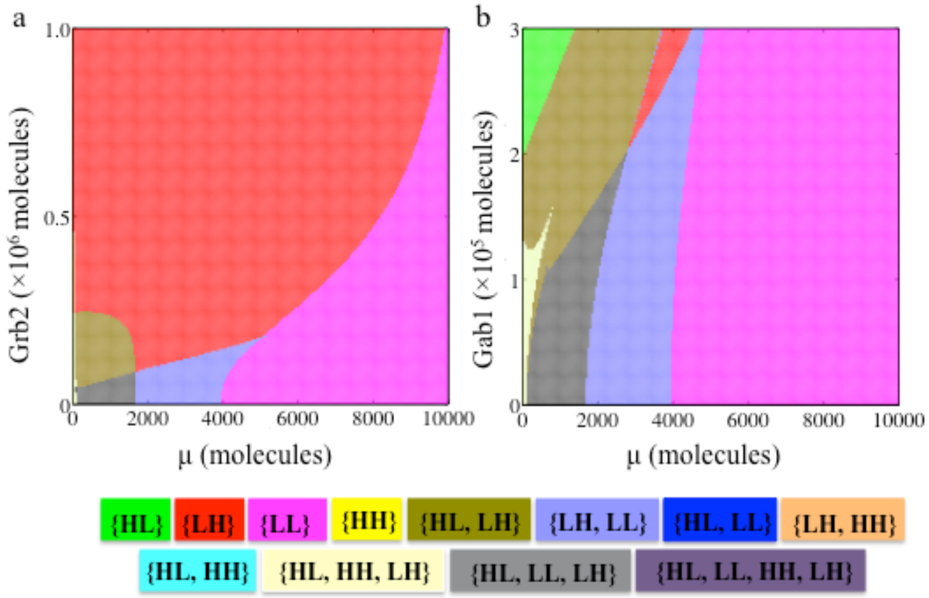

Supplementary Figure S8. Two-parameter bifurcation diagram for signal μ and signals from c-Met pathway. (a) Diagram for μ and Grb2, which could activate Rac1 protein. (b) Diagram for μ and Gab1, which could activate both Rac1 and RhoA. The colors for different phases are shown at the bottom.

References

1. Guilluy, C., Garcia-Mata, R. & Burridge, K. Rho protein crosstalk: another social network? *Trends Cell Biol.* **21,** 718–726 (2011).
2. Huang, B. *et al.* The three-way switch operation of Rac1/RhoA GTPase-based circuit controlling amoeboid-hybrid-mesenchymal transition. *Sci. Rep.* **4,** (2014).
3. Lu, M., Jolly, M. K., Levine, H., Onuchic, J. N. & Ben-Jacob, E. MicroRNA-based regulation of epithelial-hybrid-mesenchymal fate determination. *Proc. Natl. Acad. Sci.* **110,** 18144–18149 (2013).
4. Yamamura, S. *et al.* MicroRNA-34a suppresses malignant transformation by targeting c-Myc transcriptional complexes in human renal cell carcinoma. *Carcinogenesis* **33,** 294–300 (2012).
5. Kim, D. *et al.* MicroRNA-34a modulates cytoskeletal dynamics through regulating RhoA/Rac1 cross-talk in chondroblasts. *J. Biol. Chem.* **287,** 12501–12509 (2012).
6. Elson-Schwab, I., Lorentzen, A. & Marshall, C. J. MicroRNA-200 Family Members Differentially Regulate Morphological Plasticity and Mode of Melanoma Cell Invasion. *PLoS ONE* **5,** e13176 (2010).
7. Wang, Z., Humphries, B., Xiao, H., Jiang, Y. & Yang, C. MicroRNA-200b suppresses arsenic-transformed cell migration by targeting protein kinase Cα and Wnt5b-protein kinase Cα positive feedback loop and inhibiting Rac1 activation. *J. Biol. Chem.* **289,** 18373–18386 (2014).
8. Humphries, B. *et al.* MicroRNA-200b targets protein kinase Cα and suppresses triple-negative breast cancer metastasis. *Carcinogenesis* **35,** 2254–2263 (2014).

9. Lu, M. *et al.* Tristability in Cancer-Associated MicroRNA-TF Chimera Toggle Switch. *J. Phys. Chem. B* **117,** 13164–13174 (2013).

10. Lipshtat, A., Loinger, A., Balaban, N. Q. & Biham, O. Genetic Toggle Switch without Cooperative Binding. *Phys. Rev. Lett.* **96,** 188101 (2006).

11. Lewis, B. P., Burge, C. B. & Bartel, D. P. Conserved seed pairing, often flanked by adenosines, indicates that thousands of human genes are microRNA targets. *Cell* **120,** 15–20 (2005).

12. Friedman, R. C., Farh, K. K.-H., Burge, C. B. & Bartel, D. P. Most mammalian mRNAs are conserved targets of microRNAs. *Genome Res.* **19,** 92–105 (2009).

13. Grimson, A. *et al.* MicroRNA targeting specificity in mammals: determinants beyond seed pairing. *Mol. Cell* **27,** 91–105 (2007).

14. Garcia, D. M. *et al.* Weak seed-pairing stability and high target-site abundance decrease the proficiency of lsy-6 and other microRNAs. *Nat. Struct. Mol. Biol.* **18,** 1139–1146 (2011).
